# *Acizzia solanicola* (Hemiptera: Psyllidae) probing behaviour on two *Solanum* spp. and implications for possible pathogen spread

**DOI:** 10.1371/journal.pone.0178609

**Published:** 2017-06-02

**Authors:** Isabel Valenzuela, Piotr Trebicki, Kevin S. Powell, Jessica Vereijssen, Sorn Norng, Alan L. Yen

**Affiliations:** 1 Agriculture Victoria, AgriBio, Centre for AgriBioscience, Bundoora, Victoria, Australia; 2 Agriculture Victoria, Horsham Centre, Horsham, Victoria, Australia; 3 Agriculture Victoria, Rutherglen Centre, Rutherglen, Victoria, Australia; 4 The New Zealand Institute for Plant & Food Research Limited, Christchurch, New Zealand; 5 Agriculture Victoria, Parkville Centre, Parkville, Victoria, Australia; 6 School of Applied Systems Biology, La Trobe University, Bundoora, Victoria, Australia; USDA-ARS Beltsville Agricultural Research Center, UNITED STATES

## Abstract

Piercing-sucking insects are vectors of plant pathogens, and an understanding of their feeding behaviour is crucial for studies on insect population dynamics and pathogen spread. This study examines probing behaviour of the eggplant psyllid, *Acizzia solanicola* (Hemiptera: Psyllidae), using the electrical penetration graph (EPG) technique, on two widespread and common hosts: eggplant (*Solanum melongena*) and tobacco bush (*S*. *mauritianum*). Six EPG waveforms were observed: waveform NP (non-probing phase), waveform C (pathway phase), G (feeding activities in xylem tissues), D (first contact with phloem tissues), E1 (salivation in the sieve elements) and E2 (ingestion from phloem tissues). Results showed that *A*. *solanicola* is predominantly a phloem feeder and time spent in salivation and ingestion phases (E1 and E2) differed between hosts. Feeding was enhanced on eggplant compared to tobacco bush which showed some degree of resistance, as evidenced by shorter periods of phloem ingestion, a higher propensity to return to the pathway phase once in the sieve elements and higher number of salivation events on tobacco bush. We discuss how prolonged phloem feeding could indicate the potential for *A*. *solanicola* to become an important pest of eggplant and potential pathogen vector.

## Introduction

Piercing-sucking insects have the potential to evolve the ability to vector a pathogen [1]. This becomes important if we consider that several families of the Psylloidea (Triozidae, Liviidae, Psyllidae) are currently vectors of serious plant pathogens (*Candidatus* Liberibacter spp.) [2, 3]. In Australia there are no known records of a plant disease caused by Liberibacter species or reports of psyllids being vectors of plant pathogens. Nevertheless, psyllids have the potential to become vectors of pathogens, as psyllids and bacteria evolve i.e., by changing their host range [3]. For instance, in Africa *Trioza erytreae* Del Guercio an important vector of pathogenic Liberibacter spp., increased its host range to include *Citrus* spp. [4].

In Australia, three psyllid species associated with native Solanaceae have been described recently, namely *Acizzia solanicola* Kent & Taylor, *A*. *alternata* Kent & Taylor and *A*. *credoensis* Taylor & Kent (Hemiptera: Psyllidae), on a range of cultivated and wild Solanaceae hosts [5, 6]. An additional *Acizzia* sp. has been found on African boxthorn (*Lycium ferocissimum*) but awaits description [7]. *Acizzia solanicola* colonizes and develops on exotic plant species: eggplant (*Solanum melongena*), tobacco bush (*S*. *mauritianum*), Angel’s trumpet (*Brugmansia* sp.) and cape gooseberry (*Physalis peruviana*), which have become naturalised or are cultivated as food or ornamentals. The wild species *S*. *petrophilum*, an indigenous plant from the semi-arid regions of Australia, has been identified as the putative ancestral host of *A*. *solanicola* [6].

Eggplant can be perennial but the commercial production of eggplants in Australia is carried out in summer as an annual crop with 6900 tonnes produced in 2011 [8]. Conversely, tobacco bush is a perennial exotic plant species originating from Central America, currently present in every Australian State [9]. Although Angel’s trumpets and cape gooseberries can sustain large colonies of *A*. *solanicola* these hosts are less common than eggplant or tobacco bush, as they (Angel’s trumpet and cape gooseberries) are mostly found in home and public gardens. Thus, *A*. *solanicola* has access to widespread annual cultivated hosts such as eggplant and access to perennial hosts such as tobacco bush. Given that the tomato potato psyllid, *Bactericera cockerelli* (Šulc) (Hemiptera: Triozidae) [10], the vector of *Candidatus* Liberibacter solanacearum (CLso) [11] also colonizes eggplants and cape gooseberries, it is feasible that both psyllid species could co-exist and acquire phloem-restricted pathogens on their shared hosts. *Bactericera cockerelli* was detected in February 2017 in mainland Australia [12] posing a risk to horticultural industries [13] and both the insect and pathogen *Ca*. L. solanacearum are present in neighbouring Norfolk Island [14] and New Zealand [15].

Field and laboratory observations indicate that *A*. *solanicola* can be found on all plant parts, but has a preference for the new growth. Most adults can be found on the adaxial side of leaves mating or not mating and presumably feeding (Valenzuela pers. obs. 2016), while they prefer laying eggs on the underside of the leaves where nymphs develop (Valenzuela pers. obs. 2016).

Phytophagous hemipterans feed by inserting their stylets into plant tissues to access fluids from the mesophyll, xylem, and phloem [16]. The electrical penetration graph technique (EPG), was developed to enable researchers to determine the stylet location and associated activity in the plant tissue through detection of small changes in electrical resistance (R) and electromotive forces (emf) [17, 18]. These changes occur as a result of the mechanical effects of stylet and muscle movements which control the flow of saliva, the ingestion of plant fluids moving through the stylets and plant cell membrane potentials which derive each time a living plant cell is pierced by the insect’s stylets [19]. All these elements working independently or together produce an electrical ‘signature’ that can be correlated to the stylet tip position in the host plant tissue [19].

The EPG technique has played an important role in understanding insect-plant interactions, in particular in the study of hemipteran pests of economic importance such as aphids, leafhoppers, whiteflies and psyllids [20, 21, 22, 23]. There are several applications for the EPG technique including characterisation of the predominant feeding site i.e., xylem or phloem tissues [24], insecticide screening [25], host plant resistance screening [26], investigating the insect’s ability to vector plant pathogens [27, 28], and determining the minimum pathogen inoculation and acquisition access periods [29, 30]. Plant resistance has been assessed in cultivated and wild plants. Studies that used the EPG technique revealed that plant resistance is located in various plant tissues that range from the epidermis to the phloem. In aphids, plant resistance has been located in epidermis, parenchyma and phloem tissues as aphids spend longer times in non-probing phases and shorter times in the sieve elements on resistant cultivars of barley, soybean and apples [31, 32, 33]. In planthoppers, a particularly resistant rice cultivar (linked to the production of the secondary metabolite tricin) increased non-probing phase and decreased phloem ingestion [34]. Few studies however, have compared the effects of wild hosts on feeding behaviour by comparison to cultivated plants. In leafhoppers, feeding on non-hosts (sedge and ragweed) inhibited phloem ingestion by comparison to barley [35]. Aphids, feeding on wild *S*. *chomatophilum* and *S*. *stoloniferum* increased salivation into the phloem sieve elements, and decreased ingestion from phloem tissues indicating deterrent action of wild *Solanum* spp. [36].

Information regarding the basic biology of the newly discovered *Acizzia* species in Australia is not known. For example, it is not known if *A*. *solanicola* is a phloem feeder and if feeding behaviour differs among hots plants, particularly between wild and cultivated hosts. Yet, this aspect of psyllid biology is important to understand psyllid population dynamics in the field. Thus, our study aimed at i) confirming that *A*. *solanicola* is a phloem feeder as with other Psylloidea and ii) comparing feeding on eggplants and tobacco bush, its two most widespread hosts. We hypothesise that *A*. *solanicola* will predominantly feed from phloem tissues, as reported for other Psylloidea, and that feeding may be subject to changes depending on the host given that previous studies have identified wild hosts as having higher levels of resistance than cultivated plants [37].

## Material and methods

### Insects and plants

*Acizzia solanicola* was reared on *S*. *melongena* var. Long Purple at 20°C±2, 60%RH±10, and 14L:10D photoperiod. The colony was originally collected in January 2015 from eggplants in East Brunswick, Victoria, Australia. All insects used in the EPG recordings were young females (up to 7 days old).

Plants were grown under controlled conditions at 20°C±2, 60%RH±10, and 14L:10D photoperiod with a commercial fertilised soil potting mix (BioGrow, Dandenong, VIC). All plants used for EPG recordings had approximately 6–7 leaves. *Solanum melongena* var. Long Purple was obtained as seed stock (Mr Fothergill’s, South Windsor, NSW) and was chosen for its commercial use. Tobacco bush (*S*. *mauritianum*) seeds were collected from the field in Dorrigo West, New South Wales, Australia in April 2015. Tobacco bush seeds were pre-soaked in 500 mg l^-1^ gibberellic acid for 5 days to improve germination [38].

### Experimental procedures used in EPG assays

Feeding behaviour of *A*. *solanicola* was monitored on eggplant and tobacco bush using a GIGA-8 DC monitor with a 1-Giga Ω input resistance (EPG Systems, Wageningen, Netherlands). The signals were digitised using a DATAQ Di-700 A/D USB device card (Dataq® Instruments, Akron, OH, USA) and data were acquired and stored using Stylet+d for Windows 7 (EPG Systems, Wageningen, Netherlands). In all cases, EPG amplifiers were placed inside a purpose built Faraday cage to reduce electrical interference and the experiments took place in a climate controlled room at 22°C, under fluorescent lights. All EPG recordings were set at 100x gain.

We excised a leaf from the reared colony to collect young females of *A*. *solanicola* by carefully seizing the back of their wings with fine forceps and immobilizing them onto a stage by a gentle vacuum [39]. The immobilization allowed attachment of a gold wire (3 cm long, 18.0μm diameter) using a water-based silver conductive glue (EPG Systems, Wageningen, Netherlands). Four to six hours later (starvation period), psyllids were placed on the adaxial surface of eggplants and tobacco bush and monitored for a minimum of 8 h. The adaxial surface of the leaves was chosen as adult psyllids spend most of their time on this side of the leaf in the field and under controlled laboratory conditions (Valenzuela pers. obs. 2016). The third leaf, from the growing tip down, was used for EPG recordings as these leaves were unfolded by comparison to the new leaves from the growing tips. These leaves were still developing and thus were considered young leaves. During the first probing event the voltage was adjusted to positive output so that the output voltage during this initial phase was approximately 1V.

Once the recordings had taken place, waveform identification was performed manually using Stylet+a (EPG Systems, Wageningen, Netherlands). The number of recordings used for analyses was 23 and 20 on eggplant and tobacco bush respectively. The waveforms observed and their characteristics are described in Table 1. The program Stylet+a was developed for aphids and does not have the D waveform. We used the E1e waveform as a substitute for D. Furthermore, we did not analyse: Z waveforms related to electrical signals derived from the insect walking activities [38], A waveforms related to initial stylet penetration and salivation and, B waveforms related to epidermis/mesophyll sheath salivation [40, 41]. Therefore, our NP waveform analyses included Z waveforms and our C waveform analyses included A and B waveforms. Furthermore, the full 8 h recording was scored from beginning to end, which included the artificially interrupted waveforms at the end of the recording.

**Table 1:** Summary of main characteristics of *Acizzia solanicola* EPG waveforms and putative correlations of stylet tip position in plant tissue.

EPG waveform	Frequency (Hz)^1^	Voltage level	Stylet tips in plant tissue^3^	Putative activity^3^
**NP**	n/a	Extracellular	Not in plant tissue	Non-probing
**C**	15–19	Extracellular	Parenchyma	Salivary sheath secretion and other stylet pathway activity
**G**	6–7	Extracellular	Xylem	Ingestion
**D**	1 and 4^2^	Extracellular to intracellular	Phloem	First contact with sieve elements
**E1**	5–9	Intracellular	Phloem	Salivation
**E2**	6–10	Intracellular	Phloem	Ingestion

^1^ Frequency is shown as a range of values from 12 (G waveform) and 23 (C, D, E1 and E2 waveforms) insects on eggplants and was calculated based on the average of 3 observations/insect/waveform in different points of an 8 hr recording for each waveform.

^2^ Each waveform D changed frequency from 4 Hz at the beginning of the waveform to 1 Hz at the end of the waveform.

^3^ The position of the stylet tips in plant tissue and the probing activity was not determined in this study. Data shown were extracted from [23].

The overall characteristics of the waveforms were described based on the frequency (Hz) and, the voltage level (positive or negative). Amplitude and main electrical origin of the waveforms; resistance (R) or electromotive (emf) were not correlated with specific behaviors for this insect in this study. Frequency was calculated manually based on the average of 3 observations/insect/waveform chosen randomly in different points of the 8 h recording for each waveform on eggplant. Not all insects displayed all the waveforms thus, the number of observations/waveform varied depending on the number of psyllids that displayed that particular waveform. For instance, of the 23 psyllids on eggplant 12 displayed the G waveform while all the other waveforms were displayed by all 23 psyllids.

Selected sequential and non-sequential variables (i.e. information inherent to or irrespective of waveform order respectively) were calculated using Excel version 4.4.3 [42] and were used for the comparative analysis of host-related feeding differences. Non-sequential variables were calculated as i) percentage probing time spent in each waveform type (waveforms C, D, E1, sustained E2, E2 and G), ii) the mean number of waveforms produced per psyllid (waveforms NP, C, D, E1, single E1s, E2 and G), iii) the mean duration of each waveform per psyllid (waveforms NP, C, D, E1, E2 and G), and iv) the total duration of waveforms E1 and E2. Along with the non-sequential variables we calculated the sequential variables v) the number of probes before and after the 1^st^ E1, vi) the time to 1^st^ probe from start of EPG, vii) the time from 1^st^ probe to 1^st^ E1/E2, and viii) the time from 1^st^ probe to 1^st^ sustained E2. We also recorded the number of insects that produced a particular waveform on both hosts.

The choice of EPG variables was based on previous EPG work that multiple hosts [25, 35, 36] as we have done in this study.

### Statistical analyses

All non-sequential and sequential variables (based on 8 h recording periods) were initially plotted using boxplots with groups (Eggplant and Tobacco bush) to illustrate any potential treatment differences, complementing the formal analyses to follow. These variables were then analysed using analysis of variance (ANOVA) [43] for continuous variables and non-parametric Kruskal-Wallis ANOVA [43] for discrete, count data.

For ANOVA analyses, *Plant* (with two levels: eggplant and tobacco bush) was modelled as the Treatment structure. There was no block structure used. All residual values were examined graphically to ensure normality and homogeneity of variances. Observations with standardised residuals greater than 3.0 were excluded from analyses. Fishers protected Least Significant Difference (LSD) test (at 0.05 level of significance) were used to separate means (eggplant versus tobacco bush) when F-tests were significant, where LSD is the minimum difference between two treatment means that is statistically different at the defined level of significance (0.05). To check the F-probabilities reported in the ANOVAs, a randomization test with 4999 iterations were also performed. Where appropriate, non-sequential and sequential parameters were transformed (log transformed) to homogenize variances and normalize the data.

For Kruskal-Wallis ANOVA analyses, *Plant* was modelled as the Group factor and ranks were calculated for eggplant and tobacco bush. The differences between the ranks for the two groups were then compared using the test statistic H. The test statistic *H*, is formed by ranking the combined data set, then considering the sum of these ranks within each sample. When there are at least five cases in each of the samples, *H* has approximately a Chi-square distribution on *K*-1 degrees of freedom, where K is the sample size. We also calculated the probability of one waveform occurring after another as in [23].

All plots were created using the Lattice package in R [44]. All statistical analyses were performed in GenStat 18^th^ Edition [45].

## Results

### Overall characteristics of EPG recordings

*Acizzia solanicola* showed six distinctly different EPG waveforms on eggplant and tobacco bush. We compared and used the same waveform terminology as observed in previous studies carried out on Asian citrus psyllid, *Diaphorina citri* Kuwayama [23] and *B*. *cockerelli* [46]. Waveform frequency (Hz), voltage level and correlations of stylet tip in plant tissue are shown in Table 1.

**Fig 1:**
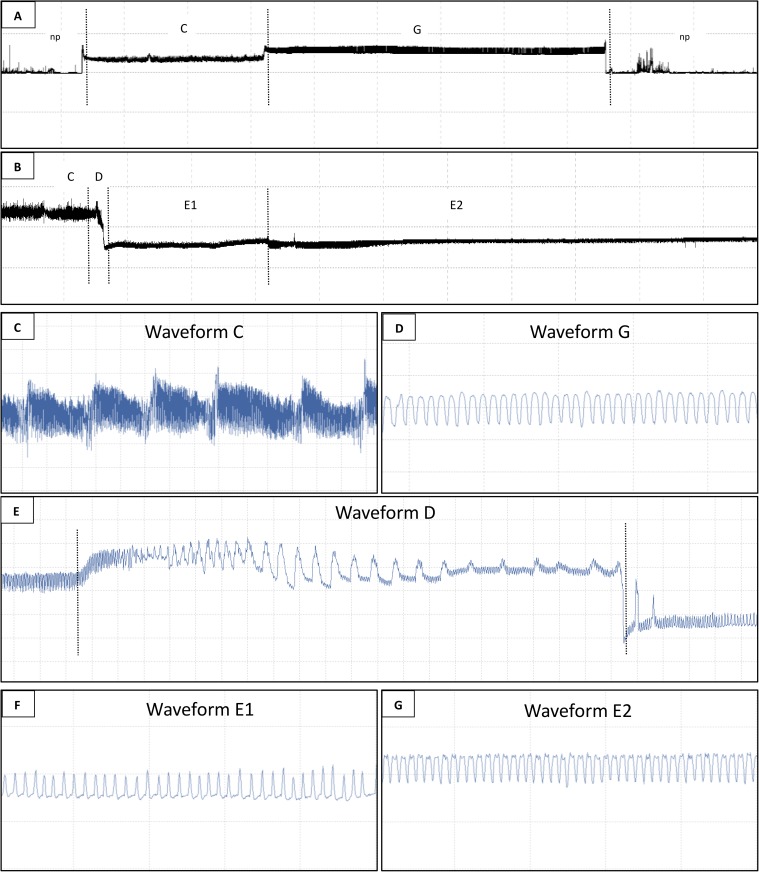
Waveforms produced by *Acizzia solanicola* on eggplant. (A) overview of 1 h recording period showing non-penetration (NP) and waveforms C and G. (B) overview of 1 h recording period showing waveforms D, E1 and E2. (C) traces of waveform C in 15 s overview. (D) traces of waveform G in 5 s overview. (E) traces of waveform D in 30 s overview. (F) traces of waveform E1 in 5 s overview. (G) traces of waveform E2 in 5 s overview. Refer to Table 1 for explanation of putative stylet tip position in plant tissue for each waveform.

Waveforms NP (non-probing), C (pathway), D (intracellular activity in the sieve elements), E1 (salivation into sieve elements), E2 (ingestion from phloem tissue) and G (ingestion from xylem tissue) were identical to waveforms described in previous studies (Fig 1A and Fig 1B). Traces of waveforms C, G, D, E1 and E2 are shown in (Fig 1C, Fig 1D, Fig 1E, Fig 1F and Fig 1G respectively). All the afore mentioned waveforms were seen in both hosts. No potential drop waveforms (pd) from intracellular activity in the pathway phase were observed, as is normally observed with aphids.

### Sequence of EPG waveforms on eggplant and tobacco bush

**Fig 2:**
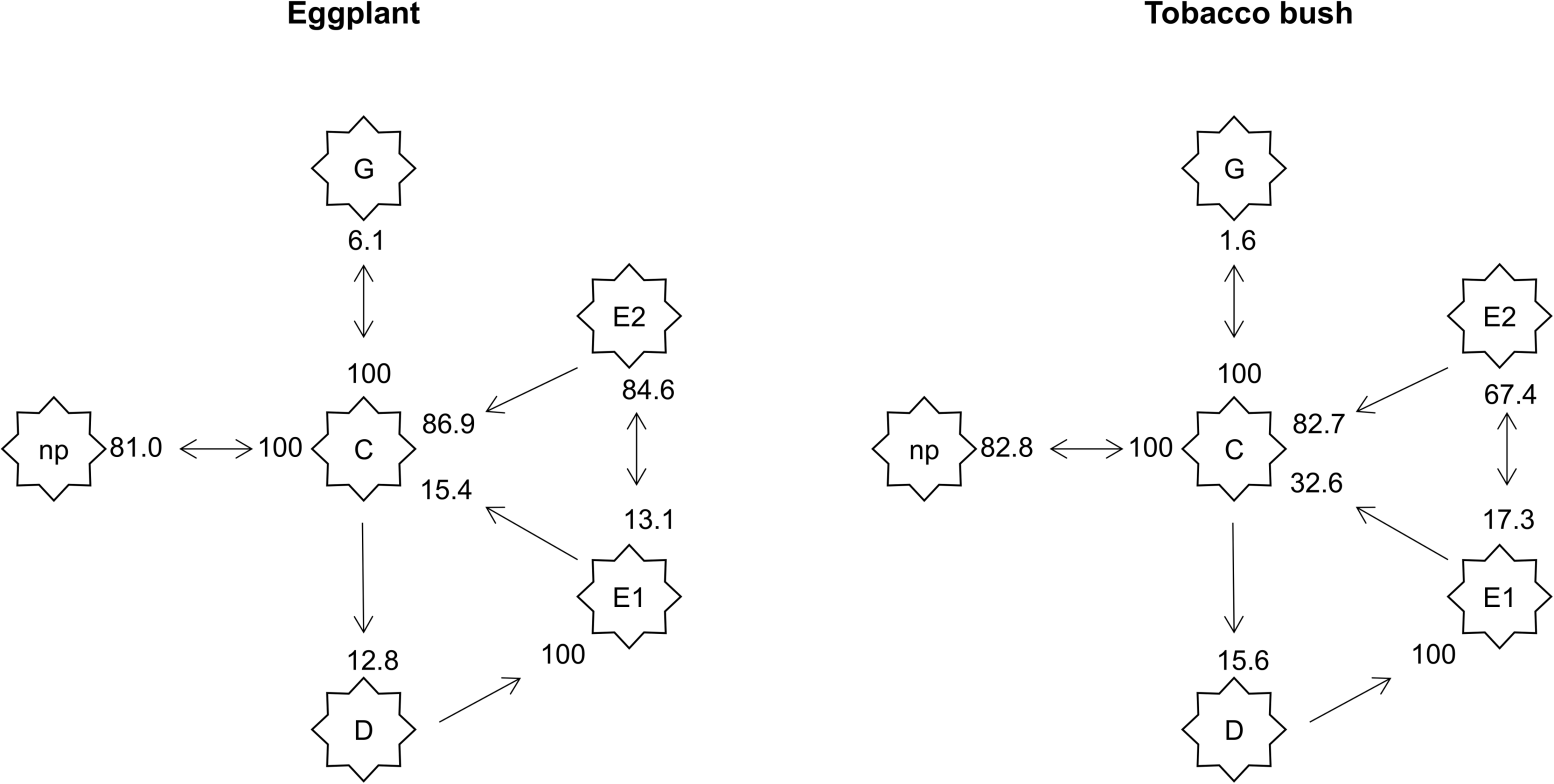
A kinetogram of *Acizzia solanicola* probing behaviour on eggplant and tobacco bush. Waveform types are enclosed in star symbol while values near the arrows are the probabilities of one waveform occurring after another (n = 23 and n = 20 on eggplants and tobacco bush respectively).

The general sequence of waveform events was the same on both hosts and was similar to previous studies on *D*. *citri* [23], but the probability of a particular waveform occurring after another was for particular waveform sequences different between hosts (Fig 2). For all psyllids used in this study, the probing sequence always started with C, the intercellular pathway phase. Once in C, psyllids preferred to return to the non-probing phase (NP) than to initiate xylem feeding (G) or phloem contact (D) (Fig 2). There was approximately 82% chance of returning to the non-probing phase (average between the two hosts), and 14% of initiating phloem contact on both hosts (average between the two hosts) (Fig 2). There was however, a difference in the probability of initiating xylem feeding which was four times higher on eggplant than on tobacco bush (6.1% and 1.6% respectively) (Fig 2). Once in E1, psyllids were more likely to initiate phloem sap feeding on eggplant than on tobacco bush (84.6% vs 67.4%) and, were less likely to revert to C on eggplant than on tobacco bush; the probabilities of reverting to C from E1 was 15.4% and 32.6% on eggplant and tobacco bush respectively (Fig 2). Salivation events occurred while ingesting phloem and were less prevalent in eggplant than tobacco bush (13.1% vs 17.3%). Overall, it is assumed that *A*. *solanicola* had some type of impediment to feed from phloem tissues on tobacco bush as they reverted more often to the pathway phase once in E1.

### Probing behaviour on eggplant and tobacco bush

Waveforms NP (non-probing), C (pathway), D (first contact with sieve elements), E1 (salivation), sustained E2 (E2> 10 min) and E2 (phloem ingestion) appeared in almost all EPG recordings ((Table 2), while waveform G (ingestion from xylem tissue) appeared less frequently with 52% and 45% of psyllids showing xylem ingestion on eggplant and tobacco bush respectively (Table 2).

**Table 2:** Number of *Acizzia solanicola* that produced a particular waveform type (n = 23 and n = 20 on eggplant and tobacco bush respectively).

Number (percentage) of *A*. *solanicola* that produced each waveform type						
Host	NP	C	D	E1	E2	G
Eggplant	23/23 (100)	23/23 (100)	23/23 (100)	23/23 (100)	23/23 (100)	12/23 (52)
Tobacco bush	20/20 (100)	20/20 (100)	19/20 (95)	19/20 (95)	19/20 (95)	9/20 (45)

**Fig 3:**
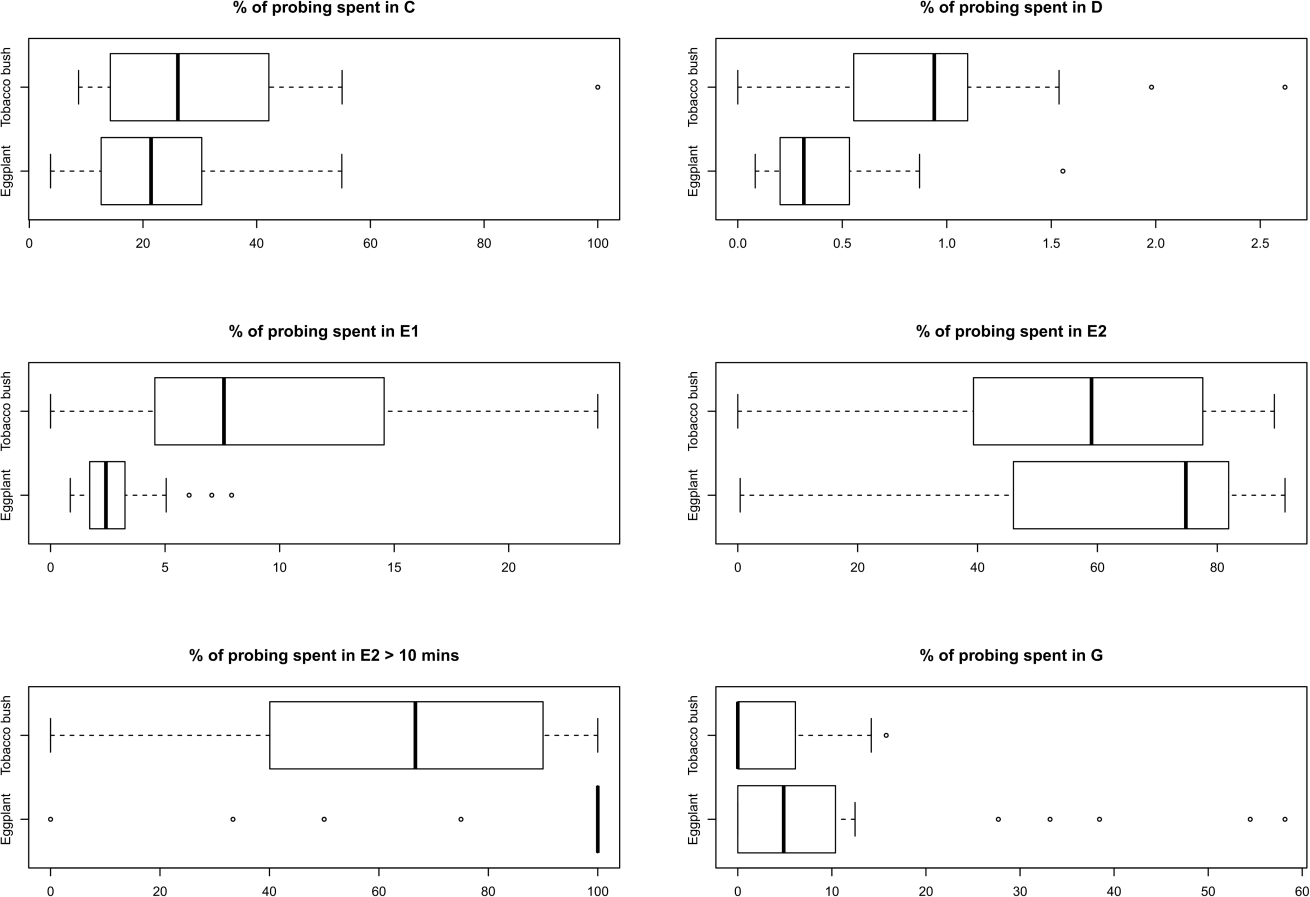
Boxplots representing percentage probing spend in each waveform of *A*. *solanicola* (n = 23 and n = 20 on eggplants and tobacco bush respectively).

Percentage probing spend in C was not different between hosts but percentage probing spend in waveforms D, E1 and sustained E2 was significantly lower on eggplant for each waveform (Table 3). For instance, probing time spend in D was 0.4% and 0.9%, percentage probing spend in E1 was 2.9% and 9.0% and percentage probing spend in sustained E2 was 87.3% and 63.2%, on eggplant and tobacco bush for each waveform respectively. Overall percentage probing in phloem ingestion E2 and xylem ingestion G was not different between hosts (Table 3). All comparisons were significant except for C, E2 and G which were not different between hosts (Table 3). Boxplots are presented that show the data used to calculate percentage probing spend in each waveform (Fig 3).

**Table 3:** Percentage probing spend in EPG waveforms of *Acizzia solanicola* on eggplant and tobacco bush (mean, n = 23 and n = 20 on eggplant and tobacco bush respectively).

Percentage of probing spend in each waveform type						
Host	C	D	E1^1^	E2	Sustained E2^2^	G^1^
Eggplant	22.3	0.4	2.9 (15.3)	62.8	87.3	11.5 (23.7)
Tobacco bush	30.9	0.9	9.0 (29.7)	55.6	63.2	3.6 (20.0)
LSD (5%), (H-statistics)	11.00	0.29	14.06	16.20	18.10	0.92
F-test probability, (Chi-square probability)	0.120	0.001	<0.001	0.371	0.010	0.301

^1^ Kruskal-Wallis one-way ANOVA was calculated for variables percentage of probing spend in E1 and G. In these two cases we report the raw mean followed by the mean rank in parentheses, as well as H-statistics and Chi-square probability.

^2^ sustained E2 is E2>10 min

**Fig 4:**
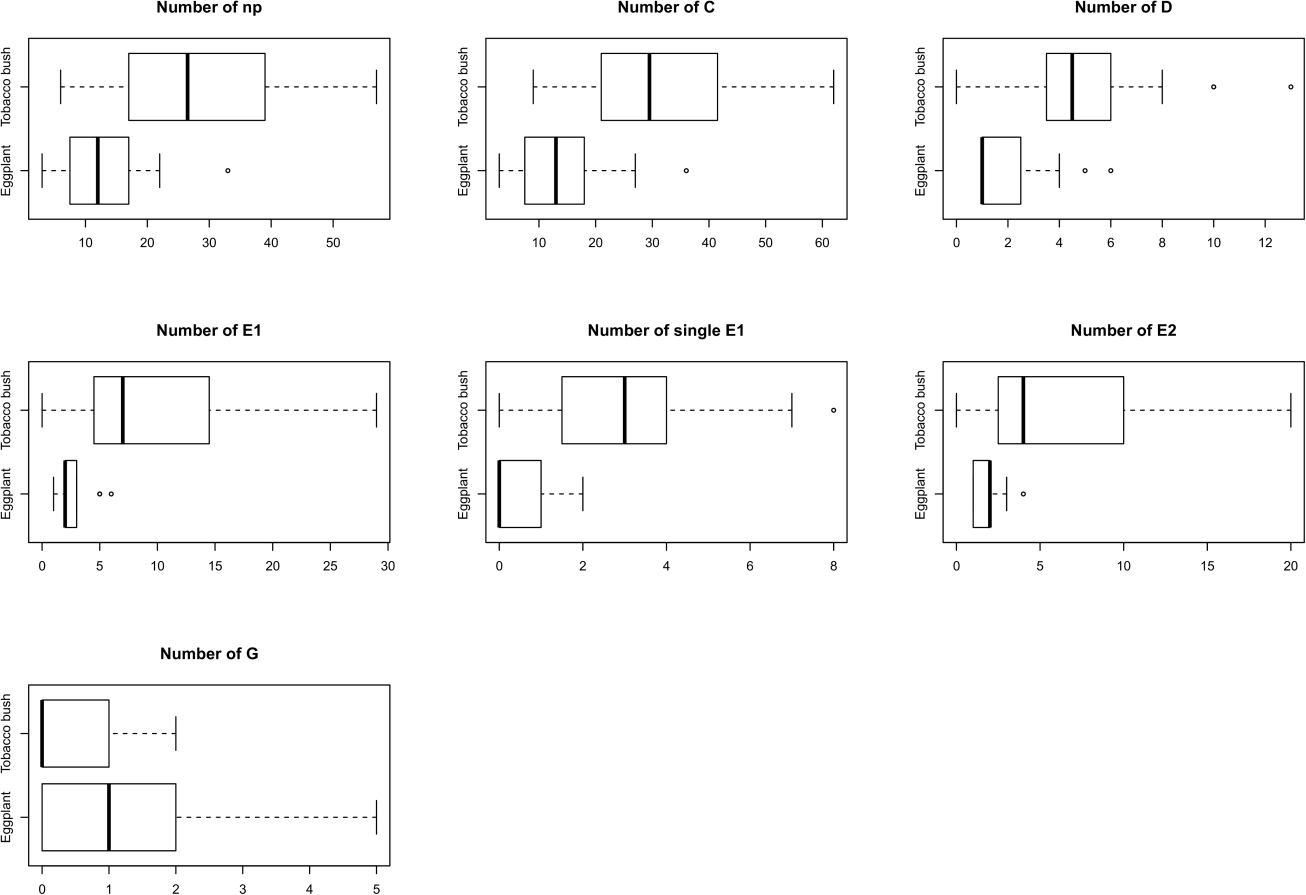
Boxplots representing number of waveforms of *A*. *solanicola* (n = 23 and n = 20 on eggplants and tobacco bush respectively).

We calculated additional non-sequential variables such as the mean number of waveform events and their duration (Table 4 and Table 5). The mean number of waveform events was lower and significantly different on eggplant for all waveforms except the xylem ingestion phases (G) (Table 4). This also included the number of single E1s (E1s not followed by E2) which were 10 times higher on tobacco bush by comparison to eggplant (Table 4). Boxplots are presented that show the data used to calculate number of EPG waveforms (Fig 4).

**Table 4:** Number of EPG waveforms of *Acizzia solanicola* on eggplant and tobacco bush (mean, n = 23 and n = 20 on eggplant and tobacco bush respectively).

Number of waveform events per psyllid^1^							
Host	NP	C	D	E1	Single E1^2^	E2	G
Eggplant	12.6 (15.2)	13.9 (14.8)	2.0 (14.8)	2.4 (14.1)	0.3 (13.4)	1.9 (16.1)	1.0 (23.6)
Tobacco bush	28.2 (29.9)	32.1 (30.2)	5.3 (30.2)	9.8 (31.1)	3.1 (31.9)	6.3 (28.8)	0.6 (20.2)
LSD (0.05), (H-statistics)	14.71	15.95	15.95	19.53	23.13	10.97	0.81
F-test probability, (Chi-square probability)	<0.001	<0.001	<0.001	<0.001	<0.001	<0.001	0.325

^1^ Kruskal-Wallis one-way ANOVA was calculated for all waveforms. We report the raw mean followed by the mean rank in parentheses, as well as H-statistics and Chi-square probability.

^2^ Single E1s are E1s not followed by E2.

**Table 5:** Duration of EPG waveforms of *Acizzia solanicola* on eggplant and tobacco bush (mean, n = 23 and n = 20 on eggplant and tobacco bush respectively).

Duration of waveform events per psyllid (seconds)^1^								
Host	NP	C	D	E1	E2	G	Total duration of E1	Total duration of E2
Eggplant	2.715 (517)	2.508 (321)	1.617 (40)	2.366 (231)	3.740 (5471)	1.690 (49)	2.693 (494)	3. 978 (9508)
Tobacco bush	2.585 (383)	2.210 (161)	1.438 (27)	2.138 (137)	3.180 (1510)	1.310 (20)	3.004 (1009)	3.814 (6523)
LSD (0.05), (H-statistics)	0.244	0.116	0.162	0.236	0.453	0.993	0.35	0.46
F-test probability, (Chi-square probability)	0.287	<0.001	0.031	0.058	0.017	0.446	0.083	0.481

^1^ All values were log transformed for analysis of variance. The values given are the log transformed means followed by the back-transformed means in parentheses.

**Fig 5:**
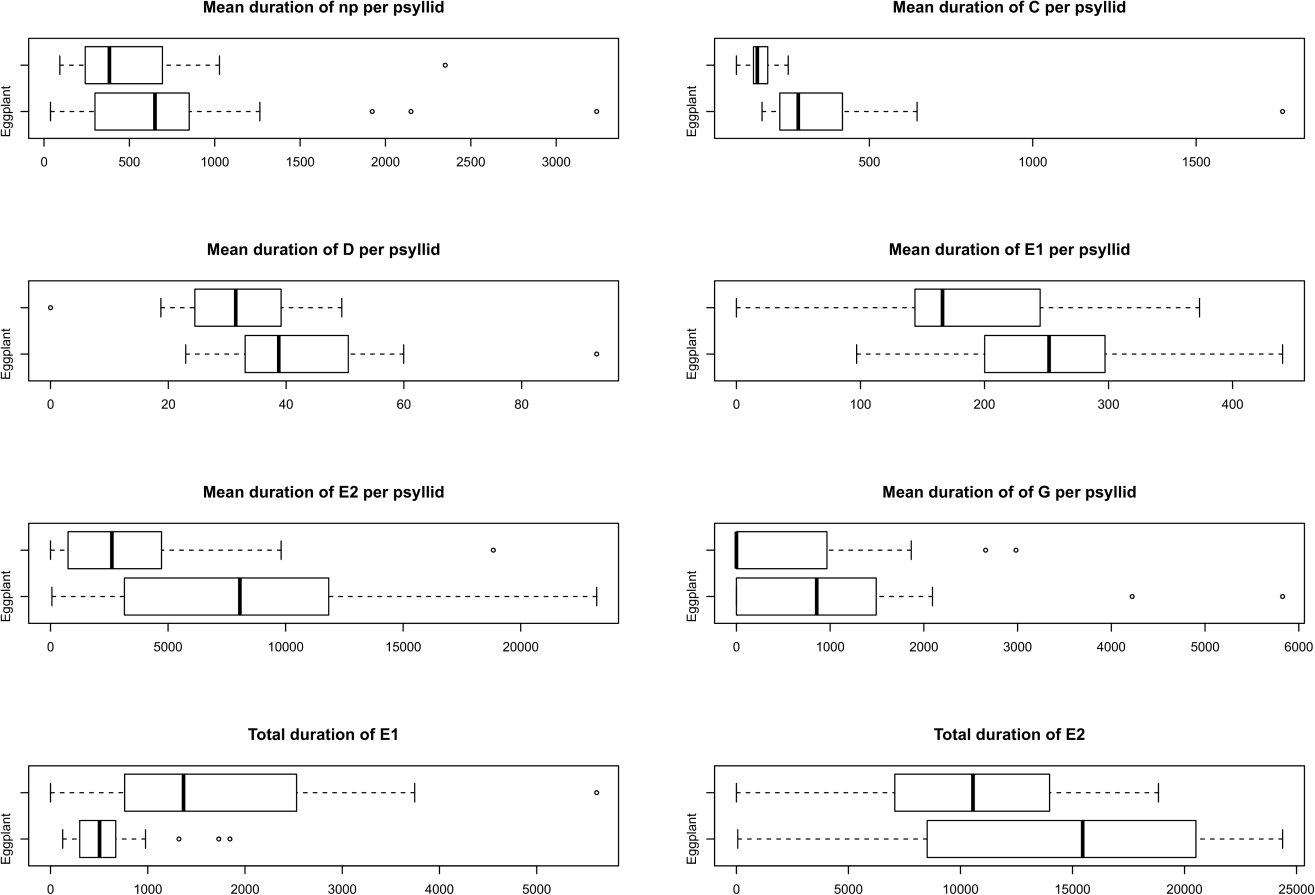
Boxplots representing duration of waveforms of *A*. *solanicola* (n = 23 and n = 20 on eggplants and tobacco bush respectively).

Mean duration per psyllid was also significantly different between hosts for almost all waveform types except for the non-probing (NP), salivation (E1) and xylem ingestion phases (G) (Table 5). Mean duration per psyllid was significantly higher on eggplant than tobacco bush for waveform C, D, and E2 (Table 5). Additional EPG variables were measured in this study such as the total duration of E1 and E2 (Table 5). Total duration of E1 was 10 min (range 2–31 min) and 30 min (range 6–94 min), and total duration of E2 was 234 min (range 1–406 min) and 175 min (range 73–314 min) for each variable on eggplant and tobacco bush respectively (Table 5) (Note that data in Table 5 are shown in seconds). Boxplots are presented that show the data used to calculate duration of EPG waveforms (Fig 5).

**Fig 6:**
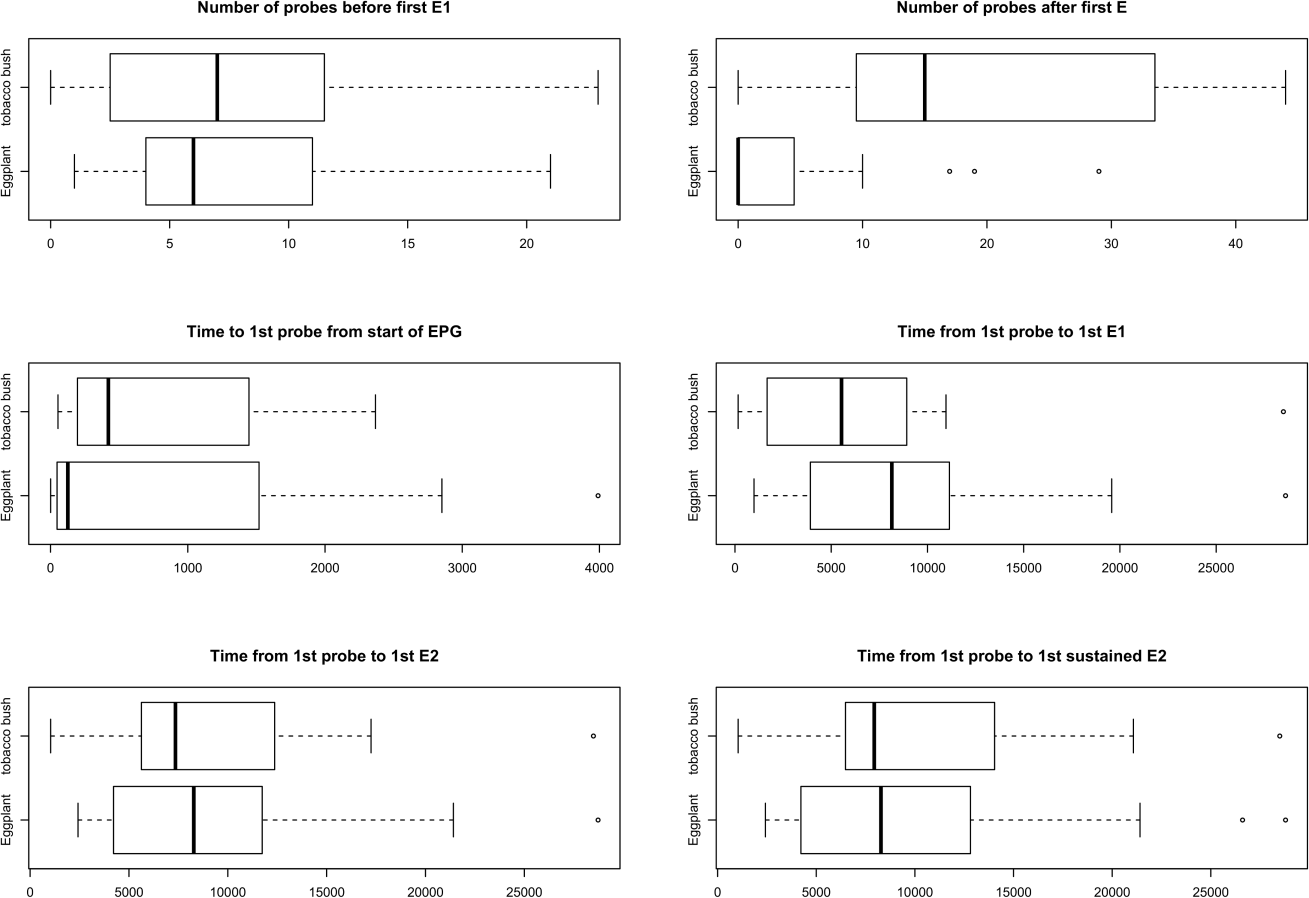
Boxplots representing sequential waveforms of *A*. *solanicola* (n = 23 and n = 20 on eggplants and tobacco bush respectively).

The analysis of sequential variables revealed that there were the same number of probes before the 1^st^ E1 on both hosts (8 probes on average approximately), but that there were significantly fewer probes after the 1^st^ E1 on eggplant. For instance, there were 4 probes on eggplant and 19 probes on tobacco bush (Table 6). Interestingly, we found that the time elapsed to reach the sieve elements (Time from 1^st^ Probe to 1^st^ E1, 1st E2 and 1^st^ sustained E2) was higher on eggplants than tobacco bush but was not statistically different (Table 6). Time to 1^st^ probe from start of EPG was 14 min and 13 min on eggplants and tobacco bush respectively (Table 6) (Note that data shown in Table 6 are in seconds). Boxplots are presented that show the data used to calculate sequential EPG waveforms shown in boxplots (Fig 6).

**Table 6:** Sequential EPG parameters of *Acizzia solanicola* on eggplant and tobacco bush (mean, n = 23 and n = 20 on eggplant and tobacco bush respectively).

Sequential EPG parameters^1^						
Host	Number of probes before 1^st^ E1	Number of probes after 1^st^ E1	Time to 1^st^ probe from start of EPG (seconds)	Time from 1^st^ probe to 1^st^ E1 (seconds)	Time from 1^st^ probe to 1^st^ E2 (seconds)	Time from 1^st^ probe to 1^st^ sustained E2 (seconds)
Eggplant	8 (23)	4 (15)	2.059 (114)	3.839 (6906)	3.893 (7819)	3.908 (8089)
Tobacco bush	8 (21)	19 (30)	2.675 (474)	3.528 (3375)	3.855 (7164)	3.884 (7662)
LSD (0.05), (H-statistics)	0.27	15.95	0.54	0.29	0.20	0.21
F-test probability, (Chi-square probability)	0.600	<0.001	0.044	0.040	0.708	0.824

^1^ All values were log transformed for analysis of variance except for variables; number of probes before 1^st^ E1 and number of probes after 1^st^ E1. In these two cases a Kruskal-Wallis one-way ANOVA was conducted where we report the raw mean followed by the mean rank in parentheses, as well as H-statistics and Chi-square probability. For the remainder variables a standard ANOVA was carried out where we report log transformed means followed by back-transformed means in parentheses.

## Discussion

### Waveform characteristic and probing behaviour

The morphological characteristics of *A*. *solanicola* waveforms matched closely the morphology of previously described waveforms from important psyllid pests such as the Asian citrus psyllid, *D*. *citri* [23], the tomato-potato psyllid, *B*. *cockerelli* [46], and the pear psyllid, *Cacopsylla pyri* (L.) [47], including waveform D. This waveform has not been correlated to sheath termini in plant tissue due to its short duration but given it is always followed by E1, it is believed to represent the moment when stylets access the sieve elements [23, 46].

The predominant waveform in *A*. *solanicola* was the phloem intake phase (E2) with 63% and 56% of the total probing time spent in E2 on eggplant and tobacco bush respectively, when feeding on the adaxial side of young leaves. Meanwhile, xylem ingestion (G), occurred less frequently, with 11% and 4% of probing time spent in this waveform on eggplant and tobacco bush respectively. This suggests that *A*. *solanicola* is predominantly a phloem feeder as other Psylloidea [48, 49, 50, 51]. In this study, fewer psyllids feed from xylem tissues (12/23 and 9/20 psyllids on eggplant and tobacco bush respectively) suggesting that this activity is not as critical as feeding from the phloem tissues which was carried out almost by all psyllids (23/23 and 19/20 psyllids on eggplant and tobacco bush respectively).

That said, many factors influence feeding behaviour such as: dehydration [52], insect gut osmotic potential [53], host plant health [41], and host mechanical/chemical stimuli [22]. Another factor is the age of the leaves. Studies on *D*. *citri* have shown that adults ingested significantly more xylem from feeding on mature leaves than from new shoots [54]. We ask whether *A*. *solanicola* would maintain the proportions of xylem ingestion (G) seen here if feeding conditions changed i.e., if they fed on new shoots instead.

Our study investigated the effect of host on feeding behavior. Psyllids seemed to exhibit similar behaviour on the two hosts prior to the 1^st^ E1, and differed markedly thereafter. For instance, first probes occurred at around 13 and 14 minutes from the beginning of the EPG recording suggesting that *A*. *solanicola* settled relatively quickly on the leaf and started probing immediately on both hosts and, the number of probes was approximately the same before the 1^st^ E1 indicating that psyllids found minimal barriers (chemical and/or physical) to progress through the epidermis and parenchyma cells of the pathway phase on both hosts. But, once in plant tissue *A*. *solanicola* found some difficulty in feeding from the sieve elements on tobacco bush by comparison to eggplant as more probes were observed in this host after the 1^st^ E1.

Thus, we suspect that chemical and mechanical stimuli present in tobacco bush and not eggplant made tobacco bush more unsuitable and more resistant to psyllid feeding than eggplant. For aphids feeding on potato and other hosts, plant defences are known to be triggered by salivary proteins [55]. We propose a similar mechanism here where tobacco bush is more sensitive to psyllid salivary proteins as evidenced by i) the relatively high number of attempts to reach the sieve elements (waveform D), ii) the relatively high number of E1s and single E1s and, iii) shorter duration of all waveform types on tobacco bush. Presumably, increased level of defence responses were triggered after the first E1 and were more effective on tobacco bush than eggplant. For instance, the average number of probes after 1^st^ E1 on tobacco bush was 19 while on eggplants it was only 4 and the number of single E1s were 10 times higher on tobacco bush than eggplants. Thus, we postulate that plant defence responses were concentrated in the sieve elements and were only triggered after reaching these tissues as there were no differences in the number of probes that occurred before reaching the sieve elements.

Based on the probing times observed in our study and particularly on the longer duration of phloem ingestion, we conclude that *A*. *solanicola* has the potential to become an important pest on eggplants as higher phloem ingestion may well be related to a potential increase in the population of psyllids, pending on the quality of the phloem sap, one principal factor that affects insect development [56, 57]. This has important implications for the population dynamics of the insect. To further confirm higher population increase on eggplants compared to tobacco bush, *A*. *solanicola* life history (survival, development and fecundity) on both host is being currently examined.

### Relationship between plant pathogens and psyllid feeding behaviour

Our study revealed that *A*. *solanicola* feeds preferentially from phloem tissues on both hosts and that time spent in salivation (E1) and phloem ingestion (E2) phases were different between hosts. *Acizzia solanicola* salivation increased on tobacco bush but ingested significantly more phloem on eggplant (when examining % probing time spent in each waveform type). This is of particular interest as the changes in phloem salivation and ingestion are critical for acquisition and transmission of phloem-restricted bacteria as these are transmitted during salivation and acquired during phloem ingestion [29, 30].

Studies controlling the time insects spend in each waveform have shown that inoculation and acquisition efficiency of pathogens increases with the length of each access period; specifically the inoculation and acquisition access periods (IAP and AAP). This has been demonstrated for whiteflies [27], and psyllids [29, 30, 58]. In whiteflies transmission efficiency of the tomato yellow leaf curl geminivirus (TYLCV) ranged from 18% to 47% based on total E1 durations of 7 to 37 min [27]. In psyllids, CLso infected *B*. *cockerelli* required a minimum IAP of 2 h for efficient transmission to tomatoes [29] and between 5 min and 6 h for efficient transmission to potatoes [30, 58] respectively]. *Bactericera cockerelli* required 36 minutes for efficient acquisition of CLso from CLso-infected tomatoes [27]. In *D*. *citri*, the minimum period of E2 was 2 min when psyllids feed from *Ca*. L. asiaticus infected *Citrus reticulata* [54].

Our study did not measure directly inoculation and acquisition access periods (IAP and AAP) as pathogenic Liberibacter species are not known to occur in Australia although Clso is found in neighbouring Norfolk Island and New Zealand, and *Ca*. L. europaeus was detected in New Zealand [59]. Nonetheless important feeding variables involved in IAP and AAP were measured in this study such as the total duration of E1 and E2. The minimum periods of E1 recorded were 2 min and 6 min (average 10 min and 30 min) and, the minimum periods of E2 recorded were 1 min and 73 min (average 234 min and 175 min) for each variable on eggplant and tobacco bush respectively. By comparison to other studies, data from this study indicates that salivation and ingestion into and from the sieve elements were sufficiently long enough to support bacteria acquisition and transmission (from a mechanical point of view), particularly for eggplant.

## Conclusions

This is the first study on *Acizzia* spp., that characterizes EPG waveforms and feeding behaviour. The study showed that *A*. *solanicola* preferred to feed from phloem tissues relative to xylem tissues when feeding on the adaxial surface of young leaves of eggplants and tobacco bush and that tobacco bush exhibited a certain degree of phloem-based plant resistance. Feeding preferentially from phloem indicates that this psyllid could become a vector of phloem restricted bacteria. Thus, future work addressing this issue is needed. For instance, characterising the species microbiome from Australian and New Zealand populations of *A*. *solanicola* would clarify its status as a potential vector of important bacteria such as the pathogenic *Ca*. Liberibacter species.
